# Genetic parameters for welfare, infestation level, and inflammatory response traits in cage-free laying hens experimentally infested with northern fowl mites (*Ornithonyssus sylviarum*)

**DOI:** 10.1016/j.psj.2026.107449

**Published:** 2026-07-17

**Authors:** Elaina R. Sculley, Hayley L. Sutherland, Marisa A. Erasmus, Amy C. Murillo, Lucio F.M. Mota, Anna Wolc, Hinayah Rojas de Oliveira, Luiz F. Brito

**Affiliations:** a Department of Animal Sciences, Purdue University, West Lafayette, IN, USA; b Hy-Line International, Dallas Center, IA, USA; c Department of Animal Sciences, Iowa State University, Ames, IA, USA; d Department of Entomology, University of California, Riverside, CA, USA

**Keywords:** Northern fowl mites, Laying hens, Animal welfare, Genetic parameters, (Co)variance components

## Abstract

Northern fowl mite (NFM) infestation in laying hens remains an important economic and welfare challenge within North American cage-free egg production systems. Controlled challenge trials provide a framework for generating standardized phenotypes of host response. Therefore, our objectives were to quantify phenotypic variability and estimate (co)variance components and genetic parameters for indicators of hen welfare, mite burden, and skin response traits following an experimental NFM challenge. A total of 1030 laying hens were infested at 24 weeks of age (WOA) and evaluated at 26, 30, and 40-41 WOA. Mite burden was assessed using an ordinal mite count (MC) score ranging from 0 to 7 (> 10,000 mites). Skin inflammatory response traits included inflammation area (IA; cm²), skin inflammation score (SI), and skin lesion score (SL). Additional welfare traits were recorded using standardized scoring systems. Hens were genotyped with a 54 K SNP panel. Variance components were estimated using animal models fitted with single-trait genomic BLUP (GBLUP), and genetic correlations between traits were obtained using two-trait GBLUP implemented under a Bayesian framework. Heritability estimates for MC, SI, IA, and SL ranged from 0.04 ± 0.03 at 40 WOA to 0.10 ± 0.04 at 26 WOA; 0.04 ± 0.02 at 40 WOA to 0.07 ± 0.04 at 30 WOA; 0.08 ± 0.07 at 40 WOA to 0.11 ± 0.05 at 30 WOA; and 0.04 ± 0.03 at 40 WOA to 0.05 ± 0.03 at 26 WOA, respectively. Genetic correlations among traits varied in both magnitude and direction, indicating complex and age-dependent relationships between mite burden and host response. These results demonstrate that traits associated with the hen response to NFM infestation are measurable, heritable, and can be improved through genomic selection. These approaches have the potential to improve hen welfare and productivity while reducing the reliance on chemical interventions, thereby supporting the long-term sustainability goals of the poultry sector.

## Introduction

Poultry are among the most intensively farmed animals worldwide, and their welfare is highly influenced by species-specific needs and housing environments (Bist et al., 2024). Genetic selection implemented by breeding companies has driven major advancements in poultry production by improving growth rate, egg production, and feed efficiency, while more recently incorporating welfare-related traits such as disease resistance and overall resilience (Wolc et al., 2016; Holquinn et al., 2024; Arango et al., 2025). In laying hens, the long-term selection for egg production has resulted in commercial strains able to produce an average of 300 eggs per bird annually, compared to 40-60 eggs per year in their wildtype counterparts (Pym, 2013).

Recently, the global egg industry has been progressively shifting towards cage-free production systems, driven by consumer preferences and legislative changes in some countries (Vaarst et al., 2015; Caputo et al., 2023; Bist et al., 2024). While cage-free systems allow greater behavioral expression, they also create new management and health challenges, caused by an increased exposure to ectoparasites (Jarrett et al., 2022). Although ectoparasitic mites are present in caged systems, achieving effective long-term control has proven more challenging in cage-free environments, where increased bird-to-bird contact and environmental complexity can result in greater mite persistence and spread (Murillo and Mullens, 2017).

The northern fowl mite (**NFM**; *Ornithonyssus sylviarum*) is an obligate hematophagous ectoparasite of domestic and wild birds and an important economic pest of laying hens in North America (Axtell and Arends, 1990; Murillo and Mullens, 2017). While its life cycle, host association, and epidemiology have been extensively reviewed (Murillo and Mullens, 2020; Holquinn et al., 2024), several biological characteristics remain central to its persistence in commercial hen flocks. Briefly, NFM complete their entire life cycle on the host, primarily in the vent region, allowing infestation to establish rapidly within production systems, and control strategies have focused on synthetic pesticides (Murillo and Mullens, 2016). However, growing concerns regarding environmental impacts, chemical residues in poultry products, and the limited feasibility of treatments in cage-free systems have increased interest in alternative control strategies (Martin et al., 2012; Murillo et al., 2017; Murillo and Mullens, 2020; Holquinn et al., 2024). Effective integrated management depends on early detection and consistent monitoring, yet infestation often persists once established due to the on-host nature of the NFM (Murillo et al., 2017; McCulloch et al., 2020). These challenges have increased interest in genetic and genomic approaches to improve host response to NFM infestation in cage-free systems, where conventional control measures are less effective.

Previous NFM infestation studies have demonstrated genetic differences in host response, including variation in mite burden and skin pathology associated with major histocompatibility complex (**MHC-B**) haplotypes (Owens et al., 2009; Murillo et al., 2016; McCulloh et al., 2020; Murillo et al., 2020). For example, specific MHC-B haplotype alleles in W-36 White Leghorns have been associated with differences in mite resistance (Fulton et al., 2006; Owens et al., 2009). Notably, differences in skin inflammation were observed between birds with the mite-resistant B21 haplotype and those with the mite-susceptible B15 haplotype. This suggests that variations in immune responses to NFM infestations depend on the genetic background of individual animals (Owens et al., 2009; Murillo et al., 2016). Evidence from population genetic studies further indicates that there are genetic differences among NFM populations in different farms, with low migration rates and high genetic similarity of mites sampled years apart on the same facilities, supporting long-term persistence once infestations become established (McCulloch et al., 2020). As a result, NFM infestation in laying hens poses persistent economic and welfare challenges in poultry production systems (Holquinn et al., 2024).

Evidence of phenotypic and genetic variability in hen response traits to mite infestation, including hen anatomical characteristics, NFM sensitivity, immunological responses, and the major histocompatibility complex (Fulton et al., 2006; Murillo et al., 2016; Lukasch et al., 2017) indicate that genomic selection may be an effective strategy for genetically enhancing hen response to NFM infestation. However, despite evidence of genetic variation in NFM response, limited information exists on the genetic architecture of host response to NFM infestation. A recent study on the same experimental cage-free challenge population demonstrated substantial between-hen heterogeneity in infestation level and associated lesions, inflammatory response, and feather-related outcomes, confirming that host response to NFM is biologically meaningful at the individual level (Sutherland et al., 2026). While relatively few studies have evaluated selection strategies for ectoparasite resistance in poultry directly, similar evidence from other livestock species indicates that ectoparasite resistance traits can be heritable and practical to use for genomic selection when reference populations with reliable phenotypes are available (Cardoso et al., 2021).

The success of a breeding program depends on the identification of traits that are heritable, repeatable, and effectively capture the biological mechanisms of interest, such as the response of laying hens to NFM infestation. Therefore, the main objectives of this study were to assess phenotypic variability and estimate (co)variance components and genetic parameters for various traits related to hen’s response to NFM infestation based on experimental NFM infestation of purebred hens in a cage-free environment. We measured mite burden, skin inflammation, inflammation area, lesion severity, and welfare traits across the infestation timeline. The goal was to assess whether challenge-derived NFM response phenotypes are measurable, heritable, and genetically informative to function as candidate traits for genomic selection. This study addresses a practical breeding question with implications for long-term improvement of hen welfare and productivity, while reducing reliance on chemical interventions.

## Material and methods

All procedures involving live animals were approved by the Purdue University Institutional Animal Care and Use Committee (PACUC; Purdue University, West Lafayette, IN, USA).

### Animals and housing

This study was performed over two trials spaced one year apart to reduce carry-over effects and gather data during the same season for both trials. The data collection procedures are detailed in Sutherland et al. (2026). Briefly, 1030 purebred White Plymouth Rock one day-old laying hen chicks were used, including 400 birds in replicate one, 600 birds in replicate two, and 30 birds used as ‘amplifier’ hens between replicates. Chicks were obtained on the day of hatch from a poultry breeding company, where they were vaccinated, beak-trimmed, and processed according to standard industry practices. They were then transported to the Purdue University Animal Sciences Research and Education Center (ASREC; West Lafayette, IN, USA) and first placed in cages until 8 weeks of age (**WOA**) and then in poultry grower rooms. At 16 WOA, pullets were transferred to cage-free rooms in the ASREC layer building with 200 birds per room. Each room was divided into five pens with 40 birds each, with each pen containing enclosed nest boxes for resting and egg laying. Birds were fed standard diets that met the breeder’s recommendations with water ad libitum and were managed according to the United Egg Producers’ Guidelines (United Egg Producers, 2024). Hens remained in the floor pens until the end of the study at 40-41 WOA.

### Mite infestation

To control the timing and intensity of infestation, a ‘mite farm’ was established in a secure room within a separate facility at Purdue University’s veterinary isolation building (West Lafayette, IN, USA). The NFM colony was maintained using a small population of “amplifier” hens that were infested with mites at 18 WOA. Experimental hens were examined during welfare assessments at 20 and 24 WOA to confirm the absence of NFM before the experimental infestation at 24 WOA. Approximately 50 adult mites were transferred to the vent area of each hen. This infestation level was chosen based on previous studies demonstrating the reliable establishment of mite populations under controlled challenge conditions as reported by Mullens et al*.* (2009).

### Phenotypic data collection and trait definition

Individual-level phenotypic data were collected to define welfare and infestation related traits for genetic analyses. Welfare traits were recorded on all 1030 birds at multiple time points, and their definitions and abbreviations are shown in Table 1. These assessments were performed at 12, 16, 20, and 24 WOA, corresponding to the pre-infestation period; 26 WOA; 30 WOA, which corresponds to peak infestation; and at 40-41 WOA. Welfare traits were adapted from the Welfare QualityⓇ Assessment Protocol for Welfare QualityⓇ Network (2019) and Heerkens et al*.* (2016). These traits included beak condition (**BK**), footpad condition (**FP**), and presence or absence of toe injuries (**TS**); feather condition and cleanliness measured on the neck (**NF**), back (**BKF**), wings (**RWF** and **LWF**), tail (**TF**), and belly (**BEF**); body weight (**BW**; in kilograms); and the presence or absence of keel tip fractures (**KTF**) and keel bone deviations (**KBD**) (Sutherland et al., 2026). These welfare-related traits were measured at 12, 16, 20, 24, 26, 30, and 40-41 WOA.

**Table 1:** Summary statistics (after quality control) for traits related to hen welfare and response to northern fowl mite infestation.

Trait	Number of records	Number of unique birds	Average (±SD)	Minimum – Maximum	CV (%)
**MC26**	953	953	2.16±0.71	1–4	32.91
**MC30**	953	953	4.15±1.16	2–6	28.04
**MC40**	952	952	2.18±0.98	1–5	44.95
**SI30**	953	953	1.59±0.49	1–2	30.90
**SI40**	952	952	1.64±0.48	1–2	29.10
**IA30**	944	944	1.23±0.98	1–12.8	79.88
**IA40**	937	937	1.09±0.38	1–5.39	34.52
**SL26**	953	953	1.12±0.32	1–2	28.7
**SL30**	953	953	1.85±0.36	1–2	19.6
**SL40**	953	953	1.40±0.49	1–2	35.00
**BK**	6781	1018	1.71±0.45	1–2	26.36
**BW(kg)**	6713	1018	1.76±0.36	0.88–2.76	20.3
**FPS**	6781	1018	1.04±0.20	1–2	19.49
**TS**	6780	1018	1.03±0.17	1–2	16.95
**NF**	6780	1018	1.41±0.49	1–2	34.88
**BKF**	6781	1018	1.47±0.50	1–2	34.04
**BEF**	6780	1018	1.41±0.49	1–2	34.92
**TF**	6779	1018	1.79±0.41	1–2	22.79
**LWF**	6780	1018	1.75±0.43	1–2	24.90
**RWF**	6781	1018	1.77±0.42	1–2	23.98
**KBD**	6780	1018	1.29±0.46	1–2	35.21
**KTF**	6781	1018	1.13±0.33	1–2	29.66

^1^MC: mite count; ^2^SI: Skin inflammation; ^3^IA: inflammation area; SL: ^4^Skin lesions. The number following the abbreviations indicates the week of age at the measurement. ^5^BK: beak score; ^6^BW: body weight measured in kilograms (kg); ^7^FPS: footpad score; **^8^**TS: toe score; **^9^**NF: neck feather score; **^10^**BKF: back feather score; **^11^**BEF: belly feather score; **^12^**TF: tail feather score; **^13^**LWF and **^14^**RWF: left- and right-wing feather scores. **^15^**KBD: Keel bone damage; **^16^**KTF: Keel tip fracture.

Feather condition was assessed using a 3-point scoring system with 0 – no or very slight wear, 1 – moderately damaged feathers, and 2 – at least one featherless area > 2.5 cm^2^ (Welfare Quality^Ⓡ^ Network, 2019). Keel bones were externally examined by palpation and scored on a scale of 0 to 2; with 0 – no deviations or deformities; 1 – minor deviations, and 2 – severe deviation and pronounced deformation of the keel bone. The KTF trait similarly evaluated with palpation for the presence / absence of a tip fracture, with assessment focused on the distal tip of the keel bone.

Skin inflammation (**SI**), inflammation area (**IA**), skin lesions (**SL**), and mite count scores (**MC**) were recorded during welfare assessments at 26, 30, and 40-41 WOA (Sutherland et al., 2026). The number of mites on each hen was counted at 26 (**MC26**), 30 (**MC30**), and 40-41 (**MC40**) WOA during welfare assessments using the scoring systems proposed by Owen et al. (2009), in which 0 (no mites); 1 (1–10); 2 (11–50); 3 (51–100); 4 (101–500); 5 (501–1000); 6 (1001–10,000); and 7 (more than 10,000 mites). The SI and IA traits were assessed near the vent region, with IA measured in cm^2^ using digital calipers (6 in. 3-Mode Digital Fractional Caliper, HuskyⓇ, Bristol, TN, USA). The SI trait was categorized based on inflammatory status and was scored as 1 – active inflammation characterized by red skin with intact scabs; or 2 – recovering skin characterized by white or light pink skin without scabs or with scabs that are cracked and lifting (Owen et al., 2009; Heerkens et al., 2016). The SL trait includes wounds that are not completely healed and show in the form of punctures on the skin and are examined near the tail and belly of the birds. SL in the vent region (where mites typically feed) were scored based on the Welfare QualityⓇ Network (2019) assesment protocol where 0: none; 1: ≤ 2 cm diameter wound; and 2: > 2 cm diameter wound. Detailed descriptions of trait definitions and scoring procedures can be found in Sutherland et al. (2026).

Phenotypic data were subjected to quality control prior to the analyses. Records were screened for data entry errors, missing values, and biologically implausible observations. For continuous traits, outlier detection was performed, and observations exceeding ± 3 standard deviations from the trait mean were removed from subsequent analyses. For ordinal traits, scoring consistency and category distributions were assessed to ensure correct classification and to identify potential inconsistencies in data recording. The number of animals and observations after the data quality control are shown in Table 1.

### Genomic data

Blood samples collected from all 1030 birds at the hatchery were used for genotyping using the Axiom 54 K Single Nucleotide Polymorphism (**SNP**) panel derived from a subset of the 600 K SNP panel (Kranis et al., 2013). Before data editing and quality control there were 59,370 SNPs spread across 38 autosomal chromosomes. Genotype quality control consisted of filtering out SNPs with minor allele frequency lower than 0.01, SNP and sample call rate lower than 0.90, SNPs with a difference between the observed and expected heterozygosity frequency greater than 0.15, and SNPs with unknown genomic positions or located on non-autosomal chromosomes. After quality control, 35,057 SNPs and 1018 individuals with both phenotypic and genomic information remained for subsequent analyses.

### Estimation of (co)variance components

*Single-trait models.* Systematic effects were determined using a backward elimination procedure, retaining effects at *p* < 0.05. The significant effects included age at measurement in days as a linear covariate, contemporary group (combination of room, pen, and repetition; *n* = 28), and recorder (20 levels). The traits measured at different WOA were analyzed as distinct traits. The model used can be described as (Eq. (1)):

(1) $$\begin{array}{c} y=\text{Xb}+\text{Za}+e \end{array}$$

where **y** is the vector of phenotypic observations; **b** is the vector of systematic fixed effects, **a** is the vector of random additive genetic effects; **X** and **Z** are the incidence matrices linking systematic and random additive genetic effects to the phenotypic records, and **e** is the vector of residuals. Random additive genetic effects were assumed to follow **a** ∼ N(0, **G**σ²ₐ), where σ²ₐ is the additive genetic variance and **G** represents the genomic relationship matrix obtained according to VanRaden (2008):

(2) $$\begin{array}{c} G=\frac{{\text{MM}}^{\text{'}}}{2\;\Sigma \;p_{i}\left(1-p_{i}\right)} \end{array}$$

where **M** is the SNP marker matrix coded as 0, 1, and 2 for genotypes AA, AB, and BB and adjusted for allele frequency 2p_i_, and p_i_ is the reference-allele frequency at locus *i*. Residuals were assumed to follow **e** ∼ N(0, **I**σ²ₑ), where σ²ₑ is the residual variance and **I** is an identity matrix.

For traits with repeated records per hen (e.g., welfare traits recorded from 12 to 40-41 WOA), a repeatability model was fitted by adding a permanent environmental effect in the previous animal model (Eq. (3)):

(3) $$\begin{array}{c} y=\text{Xb}+\text{Za}+\text{Wpe}+e \end{array}$$

where **W** represents the incidence matrix linking the phenotypic records to their permanent environmental effects, and **pe** is the vector of random permanent environmental effects assumed as $\mathrm{pe}∼N(0,I\sigma_{\text{pe}}^{2})$, where $\sigma_{pe}^{2}\;$is the permanent environmental variance. Residuals were assumed to be independent across repeated records.

*Two-trait models.* Two-trait models were fitted to estimate genetic correlations between selected trait pairs based on the following model without (Eq. (4)) or with (Eq. (5)) permanent environmental effects:

(4) $$\begin{array}{c} \left[\begin{array}{c} y_{1}\\ y_{2} \end{array}\right]=\left[\begin{array}{cc} X_{1} & 0\\ 0 & X_{2} \end{array}\right]\left[\begin{array}{c} b_{1}\\ b_{2} \end{array}\right]+\left[\begin{array}{cc} Z_{1} & 0\\ 0 & Z_{2} \end{array}\right]\left[\begin{array}{c} a_{1}\\ a_{2} \end{array}\right]+\left[\begin{array}{c} e_{1}\\ e_{2} \end{array}\right] \end{array}$$

(5) $$\begin{array}{c} \left[\begin{array}{c} y_{1}\\ y_{2} \end{array}\right]=\left[\begin{array}{cc} X_{1} & 0\\ 0 & X_{2} \end{array}\right]\left[\begin{array}{c} b_{1}\\ b_{2} \end{array}\right]+\left[\begin{array}{cc} Z_{1} & 0\\ 0 & Z_{2} \end{array}\right]\left[\begin{array}{c} a_{1}\\ a_{2} \end{array}\right]+\left[\begin{array}{cc} W_{1} & 0\\ 0 & W_{2} \end{array}\right]\left[\begin{array}{c} {\mathrm{pe}}_{1}\\ {\mathrm{pe}}_{2} \end{array}\right]+\left[\begin{array}{c} e_{1}\\ e_{2} \end{array}\right] \end{array}$$

where $y_{1}$ and $y_{2}$ are the vectors of phenotypic observations for traits 1 and 2, respectively; $b_{1}$and $b_{2}$ are the fixed effects for traits 1 and 2; $a_{1}$ and $a_{2}$ are the vectors of additive genetic effects; $pe_{1}$ and $pe_{2}$ are the vectors of permanent environmental effects, and $e_{1}$ and $e_{2}$ are the vector of residual effects. The incidence matrices **X, Z**, and **W** relate **y** to **b, a**, and **pe**, respectively.

The random effects of the animals and residuals were assumed to be normally distributed, as $\left[\begin{array}{c} a_{1}\\ a_{2} \end{array}\right]∼\text{MVN}\left(0,G⊗S_{a}\right)$, $\left[\begin{array}{c} pe_{1}\\ pe_{2} \end{array}\right]∼\text{MVN}\left(0,I⊗S_{pe}\right)$ and $\left[\begin{array}{c} e_{1}\\ e_{2} \end{array}\right]∼\text{MVN}\left(0,I⊗S_{e}\right)$, where $S_{a}=\left[\begin{array}{cc} \sigma_{a1}^{2} & \sigma_{a1,2}\\ \sigma_{a1,2} & \sigma_{a2}^{2} \end{array}\right]$ is the genetic (co)variance matrix between traits, $S_{\mathrm{pe}}=\left[\begin{array}{cc} \sigma_{\text{pe}1}^{2} & \sigma_{\text{pe}1,2}\\ \sigma_{\text{pe}1,2} & \sigma_{\text{pe}2}^{2} \end{array}\right]$ is the permanent environment (co)variance matrix between traits, and $S_{e}=\left[\begin{array}{cc} \sigma_{e1}^{2} & \sigma_{e1,2}\\ \sigma_{e1,2} & \sigma_{e2}^{2} \end{array}\right]$ is the residual (co)variance matrix.

Variance components were estimated using a Bayesian framework implemented in the GIBBSF90+ software from the BLUPF90+ family of programs (Misztal et al., 2022). Gibbs sampling was performed considering 1,000,000 iterations, with a burn-in of 200,000 and sampling every 20 iterations. The convergence of the chains was verified by graphical inspection and based on the Geweke’s criterion (*p* > 0.05) using the Bayesian Output Analysis (BOA) R package (Geweke, 1992; Smith, 2007). The single-trait heritability (h^2^) estimates for single- (Eq. (6)) and repeated-record traits (Eq. (7)) were calculated as:

(6) $$h^{2}=\frac{\sigma_{a}^{2}}{\sigma_{a}^{2}\;+\;\sigma_{e}^{2}}$$

(7) $$h^{2}=\frac{\sigma_{a}^{2}}{\sigma_{a}^{2}\;+\;\sigma_{pe}^{2}\;+\;\sigma_{e}^{2}}$$

where $\sigma_{a}^{2},\;\sigma_{pe}^{2},$ and $\sigma_{e}^{2}$ were previously defined. The predefined interpretation thresholds of h² were low when h² < 0.10; moderate 0.10–0.30; and high > 0.30. For repeated-record traits, repeatability (t) was calculated to quantify the proportion of phenotypic variance ($\sigma_{P}^{2})\;$attributable to additive genetic and permanent environmental effects combined (Eq. (8)).

(8) $$t=\;\frac{\sigma_{a}^{2}+\sigma_{pe}^{2}}{\sigma_{P}^{2}}$$

The genetic correlations were computed as the covariance between traits 1 and 2 at different WOA, divided by the square root of the additive genetic variance of trait 1 multiplied by the additive genetic variance of trait 2 (Eq. (9)):

(9) $$r_{g1,2}=\frac{\sigma_{12}}{\sqrt{\sigma_{a1}^{2}*\;\sigma_{a2}^{2}}}$$

where $\sigma_{12}$ is the additive genetic covariance between traits 1 and 2, $\sigma_{a1}^{2}$ is the additive genetic variance for trait 1, and $\sigma_{a2}^{2}$ is the additive genetic variance for trait 2. The predefined interpretation thresholds of genetic correlations were low (< |0.20|; moderate (|0.30–0.50|); and high (> |0.50|).

## Results

### Descriptive statistics

**Fig. 1:**
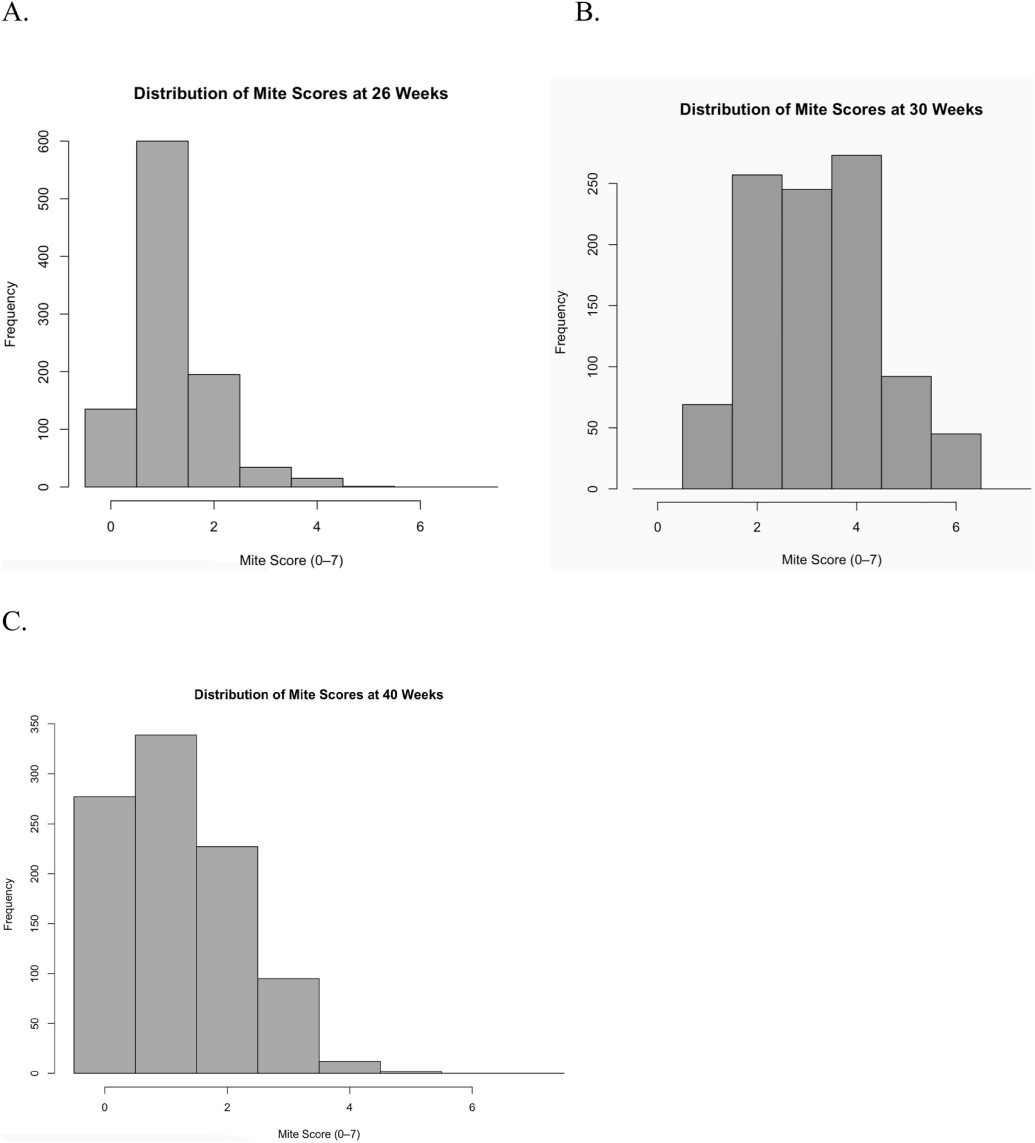
Distribution of northern fowl mite scores at 26, 30, and 40 weeks of age in laying hens experimentally infested at 24 weeks of age.

Descriptive statistics for all welfare- and NFM-related traits are shown in Table 1. At the phenotypic level, mite burden increased from 26 WOA (MC26 = 2.16 ± 0.71) to a peak at 30 WOA (MC30 = 4.15 ± 1.16) followed by a reduction at 40-41 WOA (MC40 = 2.18 ± 0.98), consistent with the expected peak-and-decline pattern of infestation following NFM challenge. The distributions of mite count scores at 26, 30, and 40-41 WOA are shown in Figure 1. Inflammation area exhibited higher variability at 30 WOA, ranging from 1 to 12.8 cm², with a CV of 79.88%, indicating strong between-hen variation in skin response to NFM infestation. Skin lesion scores were highest at 30 WOA (SL30 = 1.85 ± 0.36) and decreased at 40-41 WOA (SL40 = 1.40 ± 0.49) suggesting partial recovery over time. Overall, all traits exhibited considerable phenotypic variation, with CV varying from 16.9% for TS to 79.9% for IA30.

### Genetic parameters for NFM-infestation and related skin inflammatory response traits

Additive genetic variance was detected for NFM infestation and host response traits, including mite count (MC), skin inflammation (SI), inflammation area (IA), and skin lesions (SL) recorded at different time points (Table 2). Heritability estimates for MC, SI, IA, and SL traits ranged from 0.04 ± 0.03 (at 40-41 WOA) to 0.10 ± 0.04 (at 26 WOA); 0.04 ± 0.02 (at 40-41 WOA) to 0.07 ± 0.04 (at 30 WOA); 0.08 ± 0.07 (40 WOA) to 0.11 ± 0.05 (30 WOA); and 0.04 ± 0.03 (40-41 WOA) to 0.05 ± 0.03 (26 WOA), respectively (Table 2).

**Table 2:** Estimates of variance components and genetic parameters (posterior standard deviations) for traits related to hen response to northern fowl mite infestation.

Trait	^1^σ²ₐ	σ²ₚ	h²
MC26	0.04 ± 0.01	0.46 ± 0.02	0.10 ± 0.04
MC30	0.06 ± 0.04	1.17 ± 0.05	0.05 ± 0.03
MC40	0.02 ± 0.01	0.50 ± 0.02	0.04 ± 0.03
SI30	0.01 ± 0.005	0.22 ± 0.01	0.07 ± 0.04
SI40	0.001 ± <0.001	0.06 ± 0.002	0.04 ± 0.02
IA30	0.34 ± 0.10	3.25 ± 0.15	0.11 ± 0.05
IA40	0.01 ± 0.005	0.17 ± 0.01	0.08 ± 0.07
SL26	0.45 × 10^-2^ ± 0.003	0.09 ± 0.004	0.05 ± 0.03
SL30	0.49 × 10^-2^ ± 0.003	0.10 ± 0.004	0.05 ± 0.02
SL40	0.30 × 10^-2^ ± 0.002	0.08 ± 0.003	0.04 ± 0.03

^1^$\sigma_{a}^{2}$ refers to the additive genetic variance, h^2^ is the heritability; ^2^$\sigma_{p}^{2}$ refers to the phenotypic variance, IA: inflammation area; MC: mite count; SL: Skin lesions. The number following the abbreviations indicate the week of age at the measurement. ^3^h² is the measure of heritability for each trait.

Genetic correlations among traits varied widely in both magnitude and direction (Table 3), suggesting that the relationships between mite burden and skin response phenotypes depend on age. For the MC trait, genetic correlations were strong between nearby timepoints (MC26 vs. MC30 = 0.90 ± 0.10); (MC30 vs. MC40 = 0.97 ± 0.05) but the estimate dropped significantly as the interval between measurements increased (MC26 vs. MC40 = 0.26 ± 0.04). Among host response traits, IA showed strong genetic correlations across ages (IA30 vs. IA40 = 0.75 ± 0.20), whereas SI measured at 30 and 40-41 WOA exhibited a weak genetic correlation (SI30 vs. SI40 = 0.25 ± 0.07).

**Table 3:** Genetic correlations (± PSD) among mite count score (MC), skin inflammation status (SI), inflammation area (IA), and skin lesion score (SL) measured at 26, 30, and 40 weeks of age. Heritability (h²) estimated using the single-trait models are reported on the diagonal.

Trait	MC26	MC30	MC40	SI30	SI40	IA30	IA40	SL26	SL30	SL40
MC26	**0.10±0.04**	0.90±0.10	0.26±0.04	0.86±0.14	0.69±0.03	0.66±0.02	−0.30±0.03	0.12±0.03	0.67±0.02	−0.05±0.02
MC30		**0.05±0.03**	0.97±0.05	0.84±0.02	0.82±0.02	0.69±0.01	0.77±0.02	−0.52±0.02	0.78±0.06	0.82±0.01
MC40			**0.04±0.03**	−0.18±0.06	0.35±0.10	0.16±0.05	−0.02±0.04	−0.02±0.01	0.78±0.02	−0.11±0.06
SI30				**0.07±0.04**	0.25±0.07	0.75±0.02	0.81±0.02	−0.06±0.05	0.33±0.07	0.66±0.03
SI40					**0.04±0.02**	0.09±0.07	0.22±0.09	0.01±0.07	0.15±0.08	NC
IA30						**0.11±0.05**	0.75±0.20	0.01±0.02	0.41±0.02	0.70±0.02
IA40							**0.08±0.07**	−0.08±0.05	0.27±0.07	0.59±0.07
SL26								**0.05±0.03**	NC	0.31±0.05
SL30									**0.05±0.03**	0.22±0.08
SL40										**0.04±0.03**

^1^NC: No convergence. ^2^SI and IA traits were reported for 30 and 40 WOA only because when measured on all animals, all records were the same resulting in a CV=0%.

The MC trait exhibited the strongest correlations with inflammatory response traits during peak infestation and in later host response measurements. Specifically, MC30 was strongly and positively genetically correlated with SI at both 30 and 40-41 WOA (MC30 vs. SI30 = 0.84 ± 0.02); (MC30 vs. SI40 = 0.82 ± 0.02); IA at 40 WOA (MC30 vs. IA40 = 0.77 ± 0.02), and SL scores at both 30 and 40-41 WOA (MC30 vs. SL30 = 0.78 ± 0.06), and (MC30 vs. SL40 = 0.82 ± 0.01). On the other hand, several genetic correlations were small or close to zero, particularly those involving MC40 with IA and early SL (MC40 vs. IA40 = −0.02 ± 0.04; MC40 vs. SL26 = −0.02 ± 0.01). Additionally, MC30 was moderately and negatively correlated with early SL (SL26 =−0.52 ± 0.01), indicating that a low SL score at 26 WOA could result in more mites that are able to successfully feed, leading to a higher mite score at 30 WOA. The MC trait at 26 WOA showed a moderate negative correlation with later IA (MC26 vs. IA40 = −0.30 ± 0.03). Likewise, the genetic correlation between early MC and later SL severity was low and slightly negative (MC26 vs. SL40 = −0.05 ± 0.02), suggesting that early MC is not strongly predictive of subsequent SL severity. These results indicate that the genetic relationships between MC, IA, and SL differ across the NFM infestation timeline (26, 30, and 40-41 WOA; Table 3).

### Genetic parameters for hen welfare traits in response to NFM-infestation

Traits directly related to NFM infestation and host inflammatory response, which are included among welfare indicators used in cage-free assessments, showed measurable additive genetic variance. This indicates that they could be valuable as auxiliary traits in poultry breeding programs (Table 4). Heritability estimates for welfare-related traits ranged from low to moderate, including FP (0.01 ± 0.071) and feather scores, which ranged from 0.002 ± 0.001 (NF) to 0.03 ± 0.009 (BEF).

**Table 4:** Estimates of variance components (±PSD) for hen health and welfare-related traits in laying hens.

Trait	$\sigma_{a}^{2}$	h^2^	$\sigma_{\text{pe}}^{2}$	$\sigma_{p}^{2}$	t
BK	0.68 ± 0.16	0.22 ± 0.05	1.35 ± 0.16	3.04 ± 0.16	0.67 ± 0.02
BW	0.01 ± 0.001	0.35 ± 0.04	0.003 ± <0.001	0.02 ± <0.001	0.53 ± 0.02
FPS	0.005 ± 0.002	0.01 ± 0.007	0.001 ± <0.001	0.04 ± <0.001	0.04 ± 0.008
TS	0.003 ± 0.001	0.01 ± 0.006	0.005 ± <0.001	0.03 ± <0.001	0.03 ± 0.008
NF	0.8 × 10^-4^ ± 0.001	0.002 ± 0.001	0.001 ± <0.001	0.04 ± <0.001	0.01 ± 0.003
BKF	0.003 ± 0.001	0.02 ± 0.003	0.003 ± <0.001	0.05 ± <0.001	0.01 ± 0.005
BEF	0.002 ± 0.005	0.03 ± 0.009	0.007 ± <0.001	0.06 ± 0.001	0.05 ± 0.008
TF	0.001 ± 0.004	0.02 ± 0.006	0.007 ± <0.001	0.07 ± 0.001	0.03 ± 0.007
LWF	0.001 ± 0.001	0.01 ± 0.007	0.002 ± <0.001	0.07 ± 0.001	0.04 ± 0.009
RWF	0.001 ± 0.001	0.01 ± 0.006	0.001 ± <0.001	0.07 ± 0.001	0.02 ± 0.007
KBD	0.01 ± 0.002	0.04 ± 0.01	0.01 ± 0.002	0.19 ± 0.003	0.09 ± 0.01
KTF	0.004 ± 0.002	0.05 ± 0.01	0.004 ± 0.001	0.09 ± 0.001	0.09 ± 0.01

^1^$\sigma_{a}^{2}$ refers to the additive genetic variance,^2^h^2^ is the heritability; ^3^$\sigma_{\text{pe}}^{2}$ refers to the permanent environmental variance; ^4^$\sigma_{p}^{2}$ refers to the phenotypic variance, and ^5^t is the repeatability. ^6^BK: beak score; ^7^BW: body weight measured in kilograms (kg); ^8^FPS: footpad score; ^9^TS: toe score; ^10^NF: neck feather score; ^1^^^1^^BKF: back feather score; ^1^^^2^^BEF: belly feather score; ^1^^^3^^TF: tail feather score; ^^1^^^^4^^LWF and ^1^^^5^^RWF: left- and right-wing feather scores. ^1^^^6^^KBD: Keel bone damage; ^1^^^7^^KTF: Keel tip fracture.

Heritability estimates for skeletal damage traits (KBD and KTF) were slightly higher than those of the feather-related traits, with h^2^ estimates of 0.04 ± 0.01 for KBD and 0.05 ± 0.01 for KTF, indicating that variation in these traits is largely influenced by environmental factors, although a modest additive genetic component remains. In contrast, BW and BK showed comparatively higher heritability estimates, at 0.35 ± 0.04 and 0.22 ± 0.04, respectively, indicating stronger additive genetic control than for the other welfare-related traits.

Genetic correlations among welfare-related traits showed variability in both magnitude and direction (Table 5). Moderate and positive genetic correlations were observed between BW and BEF (0.53 ± 0.01) and between KBD and BEF (0.65 ± 0.02), suggesting that increased body weight and greater keel bone deviation were associated with more severe feather damage in the belly region. Feather score traits exhibited moderate to strong genetic relationships among them. The BKF score was positively correlated with TS (0.62 ± 0.06) and RWF (0.61 ± 0.04). Additionally, RWF and LWF were highly genetically correlated (0.91 ± 0.01), highlighting a shared genetic basis for feather coverage across the hen’s body regions. In contrast, NF showed a moderate negative genetic correlation with KTF (−0.59 ± 0.02), indicating that poor feather quality may be associated with higher KTF. BK score was genetically correlated with TS (−0.65 ± 0.02) and TF (−0.61 ± 0.01), and moderately negatively correlated with KBD (−0.35 ± 0.10), suggesting that birds with poor beak conditions may also have a reduced severity of feather damage and toe lesion issues.

**Table 5:** Estimates of heritability (diagonal) and two-trait genetic correlations (above diagonal) and their posterior standard deviations.

Trait	BW	FPS	TS	NKF	BKF	BEF	TF	RWF	LWF	KBD	KTF
**BK**	0.07±0.002	0.03±0.02	−0.65±0.02	−0.29±0.05	0.10±0.05	−0.02±0.02	−0.61±0.01	−0.20±0.03	−0.05±0.03	−0.35±0.10	0.20±0.10
**BW**	**0.35±0.04**	0.19±0.01	−0.44±0.02	−0.55±0.04	0.20±0.07	0.53±0.01	−0.29±0.01	−0.17±0.04	0.19±0.02	0.24±0.003	0.07±0.002
**FPS**		**0.01±0.007**	NC	−0.14±0.07	0.20±0.07	0.05±0.04	−0.56±0.03	−0.45±0.05	NC	NC	0.18±0.03
**TS**			**0.01± 0.006**	0.18±0.08	0.19±0.06	−0.70±0.02	0.62±0.03	−0.09±0.05	NC	0.42±0.03	NC
**NF**				**0.002±0.001**	0.24±0.07	−0.50±0.08	0.31±0.07	0.41±0.05	0.32±0.07	0.15±0.09	−0.23±0.08
**BKF**					**0.02 ± 0.003**	−0.25±0.07	0.62±0.06	0.61±0.04	0.57±0.03	NC	NC
**BEF**						**0.03±0.009**	NC	−0.48±0.04	NC	0.65±0.02	0.33±0.02
**TF**							**0.02±0.006**	0.80±0.02	0.47±0.04	−0.06±0.02	−0.59±0.02
**RWF**								**0.01±0.006**	0.91±0.01	−0.29±0.04	−0.32±0.09
**LWF**									**0.01±0.007**	−0.03±0.05	NC
**KBD**										**0.04±0.01**	−0.29±0.005
**KTF**											**0.05±0.01**

^1^BK: beak score; ^2^BW: body weight measured in kilograms (kg); ^3^FPS: footpad score; ^4^TS: toe score; ^5^NF: neck feather score; ^6^BKF: back feather score; ^7^BEF: belly feather score; ^8^TF: tail feather score; ^9^LWF and ^10^RWF: left- and right-wing feather scores. ^11^KBD: Keel bone damage; ^12^KTF: Keel tip fracture. NC: no convergence.

### Genetic correlations between welfare and mite-infestation traits

Genetic correlations between NFM infestation traits and welfare indicators are shown in Table 6. Several welfare indicators showed moderate to strong genetic associations with NFM infestation and host response traits, although some estimates were accompanied by relatively large posterior standard deviations, particularly for BK and KBD, likely reflecting the limited sample size (*n* = 1018). Because lower scores for welfare-related traits indicate better welfare status, the direction of the correlations should be interpreted accordingly.

**Table 6:** Posterior mean genetic correlations (± posterior standard deviation) between NFM infestation-related traits and welfare indicators.

Trait	MC26	MC30	MC40	SI30	SI40	IA30	IA40	SL26	SL30	SL40
**BK**	0.52± 0.20	NC	0.56±0.20	0.15±0.03	0.14±0.07	0.07±0.02	−0.25±0.06	−0.42±0.04	NC	−0.61±0.05
**BW**	0.31±0.25	−0.40±0.05	−0.11±0.05	0.15±0.06	NC	0.08±0.01	0.53±0.03	0.52±0.03	0.57±0.03	0.02±0.04
**FPS**	NC	NC	−0.19±0.06	0.62±0.04	−0.63±0.04	0.31±0.02	−0.37±0.06	0.02±0.07	NC	−0.20±0.08
**TS**	NC	−0.22±0.04	0.66±0.04	0.24±0.04	0.38±0.07	−0.14±0.07	−0.02±0.05	0.41±0.05	−0.03±0.06	−0.04±0.06
**NF**	0.34±0.13	0.16±0.07	0.03±0.09	−0.24±0.08	0.07±0.09	NC	0.03±0.07	0.05±0.07	−0.25±0.07	−0.01±0.06
**BKF**	NC	NC	−0.08±0.08	0.15±0.09	−0.25±0.08	0.50±0.04	−0.05±0.07	0.27±0.12	NC	0.15±0.12
**BEF**	NC	−0.50±0.04	−0.78±0.02	0.73±0.02	0.38±0.07	0.53±0.04	−0.41±0.05	0.36±0.08	−0.001±0.05	0.78±0.02
**TF**	0.08±0.03	−0.65±0.03	0.39±0.04	NC	NC	−0.40±0.03	−0.13±0.08	0.05±0.04	−0.27±0.06	0.34±0.06
**RWF**	NC	NC	0.26±0.09	0.35±0.07	0.08±0.09	−0.12±0.05	−0.15±0.08	−0.14±0.07	NC	NC
**LWF**	0.45±0.04	0.08±0.06	NC	0.24±0.04	0.10±0.13	NC	−0.35±0.06	0.24±0.05	NC	0.55±0.04
**KBD**	0.63±0.20	NC	0.16±0.05	0.002±0.03	0.43±0.05	0.31±0.01	0.13±0.05	−0.04±0.04	NC	−0.28±0.05
**KTF**	0.01±0.02	0.04±0.07	−0.57±0.03	−0.21±0.06	−0.70±0.03	−0.25±0.02	0.40±0.07	−0.12±0.04	−0.40±0.06	−0.50±0.06

^1^MC: mite count; ^2^SI: Skin inflammation; ^3^IA: inflammation area; ^4^SL: Skin lesions. ^5^The number following the abbreviations indicate the week of age at the measurement. ^6^BK: beak score; ^7^BW: body weight measured in kilograms (kg); ^8^FPS: footpad score; ^9^TS: toe score; ^10^NF: neck feather score; ^11^BKF: back feather score; ^12^BEF: belly feather score; ^13^TF: tail feather score; ^14^LWF and ^15^RWF: left- and right-wing feather scores; ^15^KBD: Keel bone damage; and ^16^KTF: Keel tip fracture.

The BW exhibited age-dependent genetic correlations with mite burden, with a moderate positive correlation at 26 WOA (BW vs. MC26 = 0.31 ± 0.25), followed by a moderate negative correlation at 30 WOA (BW vs. MC30 = −0.40 ± 0.05) and a weak negative correlation at 40-41 WOA (BW vs. MC40 = −0.11 ± 0.05). BW also showed moderate to strong positive genetic correlations with later inflammatory and SL-related traits, including IA40 (0.53 ± 0.03), SL26 (0.52 ± 0.03), and SL30 (0.57 ± 0.03), suggesting that genetically heavier hens tended to exhibit more severe skin responses at specific stages of infestation.

Among welfare score traits, BK exhibited moderate positive genetic correlations with MC26 (0.52 ± 0.20) and MC40 (0.56 ± 0.20), but negative correlations with SL26 (−0.42 ± 0.04) and SL40 (−0.61 ± 0.05), indicating that the genetic relationship between beak condition and infestation-related traits differed across response measures. BEF were associated with infestation-related traits, showing moderate to strong negative genetic correlations with MC30 (−0.50 ± 0.04) and MC40 (−0.78 ± 0.02), as well as strong positive correlations with SI30 (0.73 ± 0.02) and IA30 (0.53 ± 0.04). Given that higher BEF scores indicate greater feather damage, these results suggest that the genetic relationship between belly feather damage and NFM-related traits varied across the course of infestation and was particularly pronounced during peak infestation and later lesion development.

## Discussion

Experimental NFM challenge trials provide a practical framework for generating gold-standard phenotypes for both ectoparasite burden and host response under controlled infestation conditions. This is especially important for breeding applications, as measuring variation in both parasite load and host response enables the identification of new traits that could enhance resilience to NFM infestation. In this study, hens exhibited substantial phenotypic variation in mite burden across the infestation timeline, with mite counts peaking at 30 WOA followed by a reduction at 40 WOA, together with wide dispersion in IA and SL severity. This pattern of individual hen variability is consistent with previous controlled NFM challenge studies demonstrating heterogeneous host inflammatory responses and associated impacts on mite population growth and fitness (Owen et al., 2009). In cage-free production system, where NFM infestation can lead to poorer welfare and performance, these findings reinforce the need for sustainable control strategies that complement chemical interventions (Murillo et al., 2017). Although novel targeted treatments such as systemic acaricides have improved control options, their effectiveness may be limited by cost and practical challenges in large-scale cage-free systems, reinforcing the need for complementary long-term strategies such as genomic selection.

A major finding of this study is that traits related to NFM burden and host skin response exhibited measurable additive genetic variation in cage-free purebred laying hens (Table 2). Heritability estimates for MC, SI, IA, and SL were generally low to moderate, indicating that host genetics influence the variation in susceptibility and response to NFM infestation. This low magnitude level of heritability aligns with previous research on parasite load and immune traits in livestock, which are often shaped by complex interactions among host genetics, parasite exposure, and environmental factors (Cardon et al., 2011; Drobik-Czwarno et al., 2018; Hayward, 2022; Arango et al., 2025). Although the heritability estimates were not high, they support the feasibility of genetic improvement for NFM resilience (MC, SI, IA, and SL, Table 2). From a breeding perspective, even modest heritability can be useful when traits are included in selection programs, particularly for traits that are challenging or costly to measure on a routine basis and at large scale.

Among the infestation traits, mite burden (MC) measured at earlier time points (MC26 and MC30) proved particularly informative (Table 3). These early measurements showed higher heritability and strong genetic correlations with later inflammatory response traits, suggesting that they capture a biologically important stage of the host-parasite interaction. Specifically, early mite burden may reflect both host susceptibility to mite establishment and the initial effectiveness of host defense mechanisms. This interpretation is consistent with the biology of NFM infestation, because as infestation progresses, repeated feeding by these obligate blood-feeding mites in the vent region causes localized tissue damage and triggers a damage-associated molecular pattern of inflammatory responses (Julier et al., 2017), leading to skin irritation, inflammation, and lesion development (Murillo et al., 2017). As this inflammatory response intensifies, it can reduce mite feeding success, survival, and reproduction, thereby reshaping parasite population dynamics over time (Mullens et al., 2009; Owen et al., 2009). Consistent with this mechanism, greater IA has been associated with reduced mite survival and poorer offspring performance in controlled challenge studies (Mullens et al., 2009; Owen et al., 2009).

Biologically, ectoparasite establishment is shaped by host characteristics such as skin integrity, feather coverage, grooming behavior, and immune sensitivity, all of which can contribute to between-hen variation in infestation (Murillo et al., 2016). The observed genetic correlations between MC and inflammation-related traits support the hypothesis that the host immune response plays a key role in infestation dynamics. Owen et al. (2009) showed that increased host inflammatory response can impact the distance to blood vessels hindering the mites’ ability to access blood meals. Consistent with this mechanism, positive genetic correlations indicate that hens that have higher mite loads also tend to exhibit a stronger inflammatory response, as reflected by greater tissue damage at SL30. The negative genetic correlation between SI30 and MC40 (−0.18 ± 0.06) suggests that an increase in the inflammatory response at 30 WOA may contribute to reduced mite persistence at 40-41 WOA. This may indicate that inflammatory response contributes to limiting parasite persistence over time. Conversely, weaker correlations between early MC and subsequent host response could reflect time-delayed lesion development, partial recovery dynamics, or heterogeneity in the timing of host response activation across hens.

The strong genetic correlations between early mite infestation timepoints and the subsequent inflammatory response suggest that birds with greater mite infestation also tend to exhibit stronger inflammatory responses. This pattern is consistent with previous studies showing that hens with greater immune sensitivity respond to ectoparasite challenge with increased skin inflammation and lesion development (Owen et al., 2009; Murillo et al., 2020). Evaluation of welfare-related traits provides additional insights into how ectoparasite infestations impact bird health. Several welfare-related indicators, including BEF and BW exhibited moderate genetic correlations with inflammatory response traits, suggesting that the host’s response to ectoparasites may influence overall body condition. The BK score results warrant careful interpretation because beak condition can influence ectoparasite removal via grooming/preening efficiency. Prior work showed that beak condition is related to ectoparasite infestation levels and preening behavior in laying hens, highlighting that BK (and any beak-related management practice) can be biologically connected to ectoparasite dynamics rather than being an isolated welfare-only trait (Vezzoli et al., 2015). Because all hens in the present study were beak-trimmed at hatching, BK variation reflects post-trim beak condition changes over time.

The moderate to high genetic correlations observed between BEF and SI30 (0.73 ± 0.02) and IA30 (0.53 ± 0.04), indicate that feather condition is genetically associated with inflammatory response traits, suggesting that it may serve as an indirect indicator of host response to infestation. The amount of feather coverage near the vent region of the hen may influence mite survival and infestation levels (Murillo and Mullens, 2020). Birds with reduced feather coverage may provide less favorable condition for ectoparasite establishment (Jarrett et al., 2022), which is potentially a contributing factor to the negative genetic correlations observed between BEF and MC30 (−0.50 ± 0.04) and MC40 (−0.78 ± 0.02). These results highlight the potential role of physical host characteristics, such as feather coverage, that influence the ectoparasites ability to survive and reproduce. This is biologically plausible because the NFM resides primarily in the vent region of domestic laying hens where the feather microenvironment provides a favorable environment for mite proliferation. Previous work has shown that high infestation levels are concentrated primarily around the vent region and the feathers become visibly soiled or matted as mite numbers increase (Vezzoli et al., 2016; Murillo et al., 2020). However, the observed relationships between feather damage and host response traits are likely multifactorial. In addition to direct effects of NFM infestation, damaged skin or lesions may attract pecking from other birds, potentially increasing feather damage. Previous studies have demonstrated relationships between feather pecking behavior and immune characteristics, suggesting that immune function, feather condition, and social behavior may be biologically interconnected (van der Eijk et al., 2019). Therefore, the genetic correlations observed in this study may reflect a combination of host inflammatory responses, physical characteristics of the vent region, and secondary social interactions among hens.

The distinction between resistance and resilience is particularly important when considering breeding strategies for parasitic control. Resistance refers to the host’s ability to limit establishment and infestation completely (Arango et al., 2025). In contrast, resilience is the ability to tolerate infestation with reduced pathological consequences while maintaining normal functions (Laghouaouta et al., 2024). In commercial poultry production, complete resistance is often challenging due to the complexity of the housing environments and persistence of ectoparasite populations. Therefore, improving resilience may represent an important strategy that allows birds to maintain physiological functions and good welfare even in the presence of ectoparasites.

From a breeding perspective, the presence of additive genetic variation for traits related to both ectoparasite burden and host inflammatory response suggests that these traits could be incorporated into breeding programs aimed at improving individual response to ectoparasites. However, it is important to consider potential genetic relationships between infestation traits and other relevant traits (e.g., number of eggs, egg quality, feed efficiency), which will be done as a next step of this study. A limitation was that productivity measures could not be collected for individual hens because it was not possible to track individual hens’ egg production in our facilities. Selection focused solely on reduced mite infestation levels could unintentionally influence welfare-related traits if unfavorable genetic correlations exist. Therefore, evaluating the genetic correlations between infestation and production traits will be an important next step before implementing these traits into poultry breeding programs. While previous studies have demonstrated that NFM infestation negatively impacts hen physiology, integument, and egg quality at the phenotypic level (Vezzoli et al., 2016), the underlying genetic relationships between these traits remain unexplored.

The complex genetic relationships observed between MC and inflammatory response traits (IA, SI, and SL) suggest that these traits should be considered together at different WOA in breeding programs, likely as part of a selection sub-index. Selection solely on reduced inflammation without considering mite burden could inadvertently result in birds that tolerate higher ectoparasite load. Incorporating both infestation level and host-response traits into a multi-trait selection program may allow for balanced genetic improvement that enhances animal welfare and production while limiting ectoparasite burden.

Implementation in commercial breeding programs is most feasible through genomic selection rather than routine challenge phenotyping in nucleus flocks. Given the logistical and biosecurity constraints associated with experimental NFM challenge, large-scale phenotyping in elite breeding populations is impractical. Instead, ectoparasite challenge phenotypes could be collected in dedicated reference populations, potentially outside nucleus flock environments, and then linked to selection candidates through genomic predictions of breeding values. Similar strategies have been successfully applied for other hard-to-phenotype ectoparasite resistance traits in livestock (Cardoso et al., 2021). This strategy would allow selection for genetic improvement in NFM response while maintaining the productivity of commercial breeding systems.

Despite the promising results, there are also some limitations to be acknowledged. First, the population size was modest (*n* = 1030) for estimating genetic parameters for multiple traits, which may affect the accuracy of the estimates. Second, several traits were recorded as ordinal scores and at discrete assessment timepoints and they could have missed expression of certain phenotypes. A mite life cycle is around 1 week, so 5-10 weeks in between measurements is significant in terms of changes in the mite population. Therefore, using more frequent measurements, such as weekly scoring during the rise and decline phase, could better resolve temporal dynamics. Future work could strengthen biological interpretation and predictive ability by integrating additional traits such as hematological indicators of blood loss/host status, cytokine or antibody measures reflecting immune activation, and automatically recorded behavioral measures, particularly preening behavior and other grooming-related activities associated with ectoparasite defense. Behavioral measures such as dust bathing may also provide additional insight into host responses and welfare, although previous studies have reported that dust bathing is not an effective control measure for ectoparasite infestation (Vezzoli et al., 2015). These complementary traits may further improve our understanding of the host–parasite interactions influencing ectoparasites in poultry systems. While further work is needed to evaluate genomic prediction accuracy and response to selection, these findings provide valuable traits for future research. Overall, this study demonstrates that traits associated with NFM infestation and host response exhibit measurable genetic variation. Incorporating these traits into genomic selection programs may provide a sustainable strategy to improve welfare and productivity while reducing reliance on chemical interventions, ultimately supporting long-term sustainability goals of the poultry sector.

## Conclusions

Experimental NFM challenge phenotyping in cage-free hens revealed substantial individual variability in hen welfare, mite burden, and related response traits. These indicator traits of hen response to NFM infestation are heritable with substantial additive genetic variation. Genetic correlations among mite burden and skin response measures were variable and timepoint-dependent, supporting age-specific trait definitions and indicating the need for multiple-trait selection strategies that jointly consider ectoparasite load, host inflammatory response, and welfare-related traits. These results provide key information on novel traits and genetic parameters for developing genomic selection approaches with the long-term goal of improving hen welfare and reducing reliance on chemical mite control in cage-free egg production systems.

## CRediT authorship contribution statement

**Elaina R. Sculley:** Writing – review & editing, Writing – original draft, Visualization, Methodology, Investigation, Formal analysis. **Hayley L. Sutherland:** Writing – review & editing, Data curation. **Marisa A. Erasmus:** Writing – review & editing, Project administration, Funding acquisition, Conceptualization. **Amy C. Murillo:** Writing – review & editing, Resources, Methodology, Funding acquisition, Conceptualization. **Lucio F.M. Mota:** Writing – review & editing, Methodology. **Anna Wolc:** Writing – review & editing, Resources, Methodology. **Hinayah Rojas de Oliveira:** Writing – review & editing, Software. **Luiz F. Brito:** Writing – review & editing, Supervision, Project administration, Funding acquisition, Conceptualization.

## Disclosures

The authors declare the following financial interests/personal relationships which may be considered as potential competing interests: Dr. Anna Wolc is employed by Hy-Line International (Dallas Center, IA, USA). All the other authors declare that they have no known competing financial interests or personal relationships that could have appeared to influence the work reported in this paper.
